# Autistic clinical profiles, age at first concern, and diagnosis among children with autism spectrum disorder

**DOI:** 10.3389/fpsyt.2023.1211684

**Published:** 2023-08-17

**Authors:** Wen-Xiong Chen, Xian Liu, Zhifang Huang, Cheng Guo, Fangmei Feng, Yani Zhang, Yuanyuan Gao, Kelu Zheng, Jingyu Huang, Jing Yu, Wenqing Wei, Simin Liang

**Affiliations:** ^1^Department of Neurology, Guangzhou Women and Children's Medical Center, Guangzhou Medical University, Guangzhou, China; ^2^The Assessment and Intervention Center for Autistic Children, Guangzhou Women and Children's Medical Center, Guangzhou Medical University, Guangzhou, China; ^3^Department of Child Psychology, Guangzhou Women and Children's Medical Center, Guangzhou Medical University, Guangzhou, China; ^4^Division of Birth Cohort Study, Guangzhou Women and Children's Medical Center, Guangzhou Medical University, Guangzhou, China; ^5^Department of Children's and Adolescent Health, College of Public Health, Harbin Medical University, Harbin, China

**Keywords:** autism spectrum disorder, autistic clinical profiles, diagnostic age, family socioeconomic level, sex

## Abstract

**Background:**

To explore the relationship between autistic clinical profiles and age at first concern and diagnosis among children with autism spectrum disorder. The clinical profiles included the severity of autism, cognition, adaptability, language development, and regression.

**Methods:**

The multivariate linear regression model was used to examine the association of diagnostic age and first-concern age with autistic clinical profiles and with further stratification analysis.

**Results:**

A total of 801 autistic children were included. Language delay and regression were associated with earlier diagnostic age (language delay: crudeβ: −0.80, 95%CI%: −0.92–−0.68; regression: crudeβ: −0.21, 95%CI%: −0.43–−0.00) and the age of first concern of autistic children (language delay: crudeβ: −0.55, 95%CI%: −0.65–−0.45; regression: crudeβ: −0.17, 95%CI%: −0.34–−0.00). After stratification by sex, language delay tended to be more associated with the earlier diagnostic age among boys (crudeβ: −0.85, 95%CI%: −0.98–−0.72) than among girls (crudeβ: −0.46, 95%CI%: −0.77–−0.16). After stratification by maternal education level or family income level, language delay was most associated with the earlier diagnostic age in autistic children from families with higher socioeconomic levels.

**Conclusion:**

Language delay, rather than other symptoms, promoted an earlier diagnostic age. Among male autistic children or children from families with higher socioeconomic levels, language delay was most significantly associated with an earlier age of diagnosis. Cognitive delay, or adaptive delay, was associated with a later age at diagnosis and presented only in autistic children from families with lower socioeconomic levels. There may be sex or socioeconomic inequality in the diagnostic age for autistic children. More publicity and public education about the diversity of autistic symptoms are urgently needed in the future, especially for low-socioeconomic families.

## Introduction

Autism spectrum disorder is a neurodevelopmental disorder defined by social communication and interaction difficulties and restricted, repetitive behavior, interests, or activities, which typically appear in early childhood but may not manifest until social demands exceed capacity (1). The prevalence of autism has been rising significantly around the world for decades. The latest reported autism prevalence was 23.0 per 1,000 children aged 8 years in the USA (2). According to a recent meta-analysis, the prevalence of autism in China was 2.65 per 1,000 children (3). As the developmental disorder persists from childhood into adulthood, autism has given rise to substantial burdens on public health and the economy, which continue to increase (4, 5). Therefore, timely diagnosis and early intervention are vital for the prognosis and long-term improvement of autistic children (6–8). Previous studies have reported that effective intensive intervention improved the cognitive and language abilities of autistic children (8–10). In addition, early diagnosis and intervention may effectively reduce the economic and health expenditures of families and societies (11).

In recent years, increased awareness and the development of screening and diagnostic practices for autism have promoted the early identification of autistic children (12, 13). The study reported an evident decrease in the age of diagnosis for the last decades, with a mean diagnostic age of 10 ± 8 years old from 1960 to 1990 and 5 ± 3 years old from 1990 to 2005 (14). Experienced clinicians can reliably diagnose autistic children as young as 2 years old. However, some autistic children were not diagnosed until they were four or older (13, 15, 16). The notable variability of the mean or median age of identification for autism may partly be attributed to the heterogeneity of symptoms of autism (17). The heterogeneity among autistic individuals could sustain from the age of diagnosis to 6 years old and tends to be increasing in some children (18). Approximately 11–65% of autistic children have intellectual disabilities (19–21). Several autistic children showed gross motor delays, some with cognitive delays, and poor non-verbal communication (22). Approximately 10%−30% of autistic children manifested the regression, experiencing a loss of language and/or social communication around the age of two (23, 24). Previous studies have reported that autistic children with clinical profiles, including severity, intellectual disabilities, language delay, and regression were more likely to be diagnosed early (13, 17, 25–28). Daniels et al. found that the severity of the social communication and interaction impairment was associated with an earlier age of diagnosis (17). Mazurek et al. (27) also reported that lower intellectual functioning was related to an earlier diagnosis. Children with poor non-verbal communication were diagnosed at younger ages (28).

Child sex and sociodemographic factors have been focused on their association with the age of diagnosis for autism. The abovementioned factors related to the later diagnosis are modifiable or may be targeted for earlier diagnosis and intervention. The family's socioeconomic status (27, 29) or parental education level (30–32) was reported with the age of diagnosis. The lower socioeconomic or parental education level was associated with a later diagnostic age (17, 27, 29, 30, 32). Socioeconomic status or parental education level may influence access to health services and negatively impact children's access to subsequent diagnoses and interventions (29, 30). Higher socioeconomic status or better-educated parents may be more knowledgeable about navigating through the available service choices (30, 33). However, several studies have found that an earlier diagnosis of autism was not associated with socioeconomic status or parental education level (34). Child sex should also be considered an essential covariate in relation to the age of diagnosis for autism. The disproportionate ratio of males to females with autism partially depends on sex-based disparities in autism diagnoses, so females who meet the diagnostic criteria for autism might be missed (35, 36). Several studies have reported that females were later diagnosed with autism than males (37–39). Another study proposed that females are diagnosed later than males, and the time delay in referral diagnosis and evaluation for female autistic children may be due to a lack of understanding of the symptoms of female autistic children (38). There is a contradictory opinion on the role of child sex in influencing the age of diagnosis. Studies did not show that there was an association between child sex and age of first attention or age of diagnosis (40–42).

There was wide variability in findings associated with earlier or later diagnoses in previous studies, which may be attributed to the differences in study methodologies, study sizes, and the national socioeconomic development index. In addition, few studies have considered how the heterogeneous manifestation of autism interacts with child sex or family sociodemographic factors to influence the identification of autism. Therefore, the present study aims to explore whether the heterogeneous characteristics of autism are associated with the age of first concern and the age of diagnosis. We also further investigate whether the association between heterogeneous traits of autism and the age of diagnosis is influenced by child sex or family sociodemographic factors.

## Methods

### Study population

Recruitment occurred through the Neurology Department of Guangzhou Women and Children's Medical Center, which is a national children's medical center for the South Central Region of China, from January 2015 to December 2019. Autistic children were diagnosed by two independent clinicians. Informed consent was obtained from all participants included in the study. When recruited, a detailed questionnaire was collected, and the clinical phenotype of autistic children was systematically evaluated. There were main questions for parents/guardians to answer during the doctor's interview, including “When did you first become concerned about your child possibly having autism? And what was your first concern about?” and other relevant information was collected accordingly.

Autistic children were diagnosed according to the Diagnostic and Statistical Manual of Mental Disorders, Fifth Edition (DSM-5) (1). Then, the autistic children were systematically evaluated by the Autism Diagnostic Observation Schedule (ADOS) (43) and the Autism Diagnostic Interview-Revised (ADI-R) (44), which have high reliability and validity in diagnosing autistic children. In addition, children diagnosed under 2 years old would be followed up to obtain the definitive diagnosis at least until 2 years old. Children receiving a co-morbid diagnosis of autism associated with a secondary diagnosis of a neurologic, genetic, and/or metabolic disorder were excluded.

The parents provided informed consent. The study was approved by the Guangzhou Women and Children's Medical Center Ethics Committee.

### Measures

The measures below were used to systematically evaluate the clinical phenotype of children diagnosed with autism.

The Childhood Autism Rating Scale (CARS) (45) was used to evaluate the severity. First, the scores for the single items were summed together for a total score. Then, it classified the child as not autistic (below 30), mild or moderate autistic (30–36.5), or severely autistic (above 36.5) (45). The Chinese version of the Gesell Development Scale (GDS) (46, 47), previously validated and widely used in China (48), evaluated the developmental quotient (DQ) for children under 3 years of age. For children aged 3 years or older, the intelligence quotient (IQ) was assessed using the Chinese Wechsler Intelligence Scale for Children IV Version (CWISC-IV) (49, 50) or the Chinese Wechsler Young Children Scale of Intelligence-IV Version (CWYCSI-IV) (51–53). The abnormalities in DQ/IQ were deemed when the scores were below 70. The adaptive development was evaluated using the Modified Social Adaptation Scale for Infants and Junior Middle School Students (MSAS-IJM) (54, 55). The scale includes six dimensions: self-dependence, locomotion, work skills, communication, socialization, and self-management. The parents or guardians were asked to evaluate their child's skill level in the past 6 months. This scale consists of 132 items assessing the individual's personal and social competence from 6 months to 14 years old. Each item is worth one point. Thus, the total score is 132 (31 for self-dependence, 18 for locomotion, 20 for work skills, 23 for communication, 22 for socialization, and 18 for self-management). Self-dependence included skills such as drinking, getting dressed, and bathing. Locomotion involved sitting, running, and going to school. Work skills included drawing, opening a bottle, and cooking. Communication included speaking, reading, and writing. Socialization included showing an interest in others, having a preferred friend, and engaging in school activities. Self-management included doing something by oneself, taking care of elderly and younger people, saving money, and planning (54–56). The non-verbal autistic child was defined as a child with less than five spontaneous functional words (57, 58). A regressive autistic child was defined as a child who experienced a period of apparently normal development for the first one to 2 years of life, followed by an abrupt or gradual loss of previously acquired skills (59, 60).

### The interview

This was a retrospective interview. Our center is one of the national tertiary children's medical centers and accepts the referral of suspected autistic patients from secondary/first-level hospitals, kindergartens/schools, or directly from parents/guardians themselves. The parents/guardians of autistic children included in the study were interviewed by the doctor during the diagnostic procedures. At the point of the interview, the patient's parents/guardians may have some knowledge about autism already or may not. To acquire information regarding the referral reasons and the referral channels, we also asked each parent/guardian of the patients the following questions after finishing the ADIR assessment session. The interview questions were as follows: (1) When were you first concerned about your child possibly having autism? Or When did you become concerned that your child might be lagging in some dimension of neurodevelopment, or when did you become concerned about your child's developmental status and/or trajectory? And what was your first concern about? (2) Has your child officially been diagnosed with autism by the pediatrician or psychiatrist before this visit, and if so, when? (3) Which person or factor prompted your child to be referred to seek medical help? (4) Which developmentally backward behaviors of your child encouraged you to start seeking medical help? (5) Please, can you specifically tell us about your child's autistic-related symptoms? The responses above were given orally to the doctor.

### The covariate

Child sex was coded as male or female. Household income per month was coded on a 3-point scale, including <5,000 yuan, 5,001 to 9,999 yuan, and 10,000 yuan or more. Considering the high collinearity between parental and maternal education, we only adjusted the maternal education level. Maternal education was classified as high school or below, undergraduate, postgraduate, or above. In addition, information on birth order was obtained.

### Statistical analysis

Autistic children's characteristics in the study were presented. Counts and percentages were shown for categorical variables, the mean and standard deviation (SD) were presented for continuous variables with a normal distribution, and the median and interquartile range (IQR) were presented for continuous variables without a normal distribution. The multivariate linear regression model was used to examine the association of diagnostic age and first-concern age with autistic clinical characteristics such as severity, cognition, language, and regression. A series of interaction terms between autistic clinical phenotypes and sex, as well as autistic clinical phenotypes and maternal education and autistic clinical phenotypes, and family monthly income, were performed to determine if the relationship between diagnostic age and autistic clinical phenotypes differed by sex, maternal education, and family monthly income. Only there were significant interactions, stratified analyses were further conducted. The covariates to control for potential confounders included in the multiple regression model above were child sex, parity, maternal age at birth, maternal education, and family income. The linear regression model was also used to examine the association between the first concern age of the autistic children and the diagnostic age of autistic children. All analyses were performed using SAS statistical software version 9.3 (SAS Institute Inc., Cary, NC, USA). A two-tailed *P* < 0.05 was considered statistically significant.

## Results

A total of 801 autistic children were enrolled. Among these, 701 (87.52%) were male, and 100 (12.48) were female. The mean age at diagnosis was 3.11 ± 0.93 years old. The mean age of the first concern was 2.07 ± 0.75 years old. The median time interval from first concern to diagnosis was 0.88 (IQR: 0.42–1.42) years old. The mean CARS score was 33.85 ± 2.39. The mean DQ/IQ score was 33.85 ± 2.39. According to adaptive function, 104 (14.04%) children were classified as normal level, while 244 (32.93%) children were classified as mild level, 286 (38.6%) children were classified as borderline level, 95 (12.82%) children were classified as moderate level, 9 (1.21%) children were classified as severe level, and 3 (0.4%) children were classified as extreme severe level. About 308 autistic children were non-verbal, and 83 autistic children experienced regression. The characteristics of the study participants are presented in Table 1.

**Table 1:** Characteristics of the study participants.

**Characteristics**	***n***
**Sex**, ***n*** **(%)**	
Male	701 (87.52)
Female	100 (12.48)
**Race**, ***n*** **(%)**	
Han	788 (98.38)
Non-Han	13 (1.62)
Diagnostic age, mean ± SD	3.11 ± 0.93
First concern age, mean ± SD	2.07 ± 0.75
Time intervals from first concern to diagnosis, median (IQR)	0.88 (0.42–1.42)
**Births**, ***n*** **(%)**	
Single	784 (97.88)
Twin	17 (2.12)
**Delivery mode**, ***n*** **(%)**	
Vaginal Birth	471 (58.8)
Cesarean	330 (41.2)
**Parity**, ***n*** **(%)**	
1	461 (58.88)
2	269 (34.36)
≥3	53 (6.77)
**Maternal age at birth**, ***n*** **(%)**	
≤ 29	476 (59.43)
30–39	304 (37.95)
≥40	21 (2.62)
**Maternal education level**, ***n*** **(%)**	
High school or below	421 (52.56)
Undergraduate	347 (43.32)
Postgraduate or above	33 (4.12)
**Monthly income (yuan)**, ***n*** **(%)**	
< 5,000	130 (19.58)
5,000–10,000	240 (36.14)
>10,000	294 (44.28)
CARS, mean (SD)	33.85 ± 2.39
DQ/IQ score, mean (SD)	61.06 ± 12.92
**Adaptive function**, ***n*** **(%)**	
Extreme	3 (0.4)
Severe	9 (1.21)
Moderate	95 (12.82)
Borderline	286 (38.6)
Mild	244 (32.93)
Normal	104 (14.04)
**Language**, ***n*** **(%)**	
Verbal	493 (61.55)
Non-verbal	308 (38.45)
**Regression**, ***n*** **(%)**	
Yes	83 (10.36)
No	718 (89.64)

SD, standard deviation; IQR, interquartile range; IQ, intelligence Quotient; DQ, developmental quotient; CARS, childhood autism rating scale.

Table 2 showed the relation of autistic clinical phenotypes with the age of diagnosis for autistic children. DQ/IQ scores were negatively related to the age of diagnosis for autistic children (Crudeβ = −0.01, 95%CI = −0.01–−0.00). The adaptive function was negatively related to the age of diagnosis for autistic children (Crudeβ = −0.08, 95%CI = −0.12–−0.03). The worse language development was related to the earlier diagnostic age of autistic children (Crudeβ = −0.80, 95%CI = −0.92–−0.68). The regression was related to the earlier diagnosis age of autistic children (Crudeβ = −0.21, 95%CI = −0.43–−0.00). After adjusting the child sex, parity, maternal age at birth, maternal education, and family monthly income, the results remained.

**Table 2:** The correlation of autistic clinical phenotypes with the age of diagnosis for autistic children.

**Characteristic**	**Crudeβ (95%CI)**	**Adjusted β (95%CI)^a^**	**Adjusted β (95%CI)^b^**
CARS	0.02 (−0.0–0.05)	0.02 (−0.01–0.04)	0.01 (−0.02–0.05)
DQ/IQ score	−0.01 (−0.01–−0.00)	−0.01 (−0.01–−0.00)	−0.01 (−0.01–−0.00)
Adaptive function	−0.08 (−0.12–−0.03)	−0.07 (−0.11–−0.02)	−0.05 (−0.10–0.00)
Language	−0.80 (−0.92–−0.68)	−0.84 (−0.96–−0.71)	−0.85 (−0.99–−0.72)
Regression	−0.21 (−0.43–−0.00)	−0.22 (−0.44–−0.00)	−0.12 (−0.37–0.12)

OR, odd risk; CI, confidence interval; DQ, developmental quotient; IQ, intelligence quotient; CARS, Childhood Autism Rating Scale.

a Adjust for child sex, parity, maternal age at birth, maternal education.

b Adjust for child sex, parity, maternal age at birth, maternal education, family income.

Table 3 showed the relation of autistic clinical phenotypes with the age of first concern for autistic children. The worse language development was related to the earlier age of first concern for autistic children (Crudeβ = −0.55, 95%CI = −0.65–−0.45). The occurrence of regression in autistic children has related to the earlier age of first concern (Crudeβ = −0.55, 95%CI = −0.65–−0.45). After adjusting the child sex, parity, maternal age at birth, maternal education, and family monthly income, the results remained.

**Table 3:** The correlation of autistic clinical phenotypes with the age of first concern for autistic children.

**Characteristic**	**Crudeβ (95%CI)**	**Adjusted β (95%CI)^a^**	**Adjusted β (95%CI)^b^**
CARS	−0.02 (−0.04–0.00)	−0.02 (−0.04–0.00)	−0.01 (−0.04–0.01)
DQ/IQ score	0.00 (−0.00–0.01)	−0.00 (−0.00–0.01)	−0.00 (−0.00–0.01)
Adaptive function	0.02 (−0.02–0.05)	0.02 (−0.01–0.06)	0.02 (−0.01–0.06)
Language	−0.55 (−0.65–−0.45)	−0.56 (−0.67–−0.46)	−0.54 (−0.65–−0.42)
Regression	−0.17 (−0.34–−0.00)	−0.20 (−0.37–−0.03)	−0.11 (−0.31–0.08)

OR, odd risk; CI, confidence interval; DQ, developmental quotient; IQ, intelligence quotient; CARS, Childhood Autism Rating Scale.

a Adjust for child sex, parity, maternal age at birth, maternal education.

b Adjust for child sex, parity, maternal age at birth, maternal education, family income.

Table 4 presents stratified analyses according to autistic clinical phenotypes and sex in relation to diagnostic age. We evaluated whether sex is an effect modifier and further explored if there was an interaction between sex and the CARS scores on the diagnostic age (interaction *p* = 0.021). Only in autistic girls was the CARS score positively with the age of diagnosis (Crudeβ = 0.99, 95%CI = 0.02–0.16). There was an interaction between sex and the DQ/IQ scores on the diagnostic age (interaction *p* < 0.001). In autistic boys, DQ/IQ scores were negatively related to the age of diagnosis for autistic children (Crudeβ = −0.01, 95%CI = −0.01–−0.00). In autistic girls, DQ/IQ scores were negatively related to the age of diagnosis for autistic children (Crudeβ = −0.02, 95%CI = −0.03–−0.01). There was an interaction between sex and the adaptive function on the diagnostic age (interaction *p* < 0.001). The adaptive function was negatively related to the age of diagnosis in autistic boys (Crudeβ = −0.07, 95%CI = −0.12–−0.02) and autistic girls (Crudeβ = −0.08, 95%CI = −0.19–0.03). Child sex is an effect modifier on the relation between language and the age of diagnosis for autistic children (interaction *p* < 0.001). In autistic boys, the worse language development was related to the earlier diagnostic age of autistic children (Crudeβ = −0.85, 95%CI = −0.98–−0.72). In autistic girls, the worse language development was related to the earlier diagnostic age of autistic children (Crudeβ = −0.46, 95%CI = −0.77–−0.16).

**Table 4:** Stratified analyses according to autistic clinical phenotypes and sex with diagnostic age.

**Characteristic**	**Interaction *p*-value**	**Male**		**Female**	
		**Adjusted** β **(95% CI)**	***p*** **-value**	**Adjusted** β **(95% CI)**	***p*** **-value**
CARS	0.023	0.01 (−0.02–0.04)	0.614	0.09 (0.02–0.16)	0.013
DQ/IQ score	< 0.001	−0.01 (−0.01–−0.00)	0.014	−0.02 (−0.03–−0.01)	0.004
Adaptive function	0.001	−0.07 (−0.12–−0.02)	0.003	−0.08 (−0.19–0.03)	0.159
Language	< 0.001	−0.85 (−0.98–−0.72)	< 0.0001	−0.46 (−0.77–0.16)	0.004
Regression	0.106	−0.22 (−0.45–0.01)	0.062	−0.19 (−0.71–0.33)	0.476

OR, odd risk; CI, confidence interval; DQ, developmental quotient; IQ, intelligence quotient; CARS, Childhood Autism Rating Scale.

Adjust for child sex, parity, maternal age at birth, maternal education, family income.

Table 5 demonstrated stratified analyses according to autistic clinical phenotypes and maternal education in relation to diagnostic age. There was an interaction between maternal education and DQ/IQ score on the diagnostic age (interaction *p* = 0.012). Only in autistic children with high school or below maternal education, DO/IQ scores were negatively related to the diagnostic age (Crudeβ = −0.02, 95%CI = −0.03–−0.02). Maternal education was found as an effect modifier on the relation between adaptive function and the age of diagnosis for autistic children (interaction *p* = 0.008). The adaptive function was negatively related to the age of diagnosis only in autistic children with high school or below maternal education (Crudeβ = −0.10, 95%CI = −0.15–−0.04). There was an interaction between maternal education and language development on the diagnostic age (interaction *p* < 0.001). The worse language development was related to the earlier diagnostic age of autistic children with high school or below of maternal education (Crudeβ = −0.67, 95%CI = −0.84–−0.49), undergraduate of maternal education (Crudeβ = −0.96, 95%CI = −1.14–−0.78), postgraduate or above of maternal education (Crudeβ = −1.42, 95%CI = −2.03–−0.81). There was an interaction between maternal education and regression in the diagnostic age (interaction *p* = 0.005). In autistic children exposed to undergraduate of maternal education, regression was related to the earlier diagnosis age (Crudeβ = −0.51, 95%CI = −0.83–−0.18).

**Table 5:** Stratified analyses according to autistic clinical phenotypes and maternal education with diagnostic age.

**Characteristic**	**Interaction *p-*value**	**High school or below**		**Undergraduate**		**Postgraduate or above**	
		**Adjusted** β **(95% CI)**	***p*** **-value**	**Adjusted** β **(95% CI)**	***p*** **-value**	**Adjusted** β **(95% CI)**	***p*** **-value**
CARS	0.209	−0.02 (−0.17–0.13)	0.7698	−0.01 (−0.06–0.04)	0.744	0.03 (0–0.07)	0.0798
DQ/IQ score	0.012	−0.02 (−0.03–−0.02)	< 0.0001	0.00 (−0.01–0.01)	0.6155	0.01 (−0.0–0.04)	0.6495
Adaptive function	0.008	−0.10 (−0.15–−0.04)	0.0004	−0.04 (−0.12–0.04)	0.2966	0.09 (−0.22–0.40)	0.5452
Language	< 0.001	−0.67 (−0.84–−0.49)	< 0.0001	−0.96 (−1.14–−0.78)	< 0.0001	−1.42 (−2.03–−0.81)	< 0.0001
Regression	0.005	−0.01 (−0.29–0.27)	0.9487	−0.51 (−0.83–−0.18)	0.0026	0.46 (−0.89–1.82)	0.492

OR, odd risk; CI, confidence interval; DQ, developmental quotient; IQ, intelligence quotient; CARS, Childhood Autism Rating Scale.

Adjust for child sex, parity, maternal age at birth, family income.

Table 6 illustrated stratified analyses according to autistic clinical phenotypes and family monthly income in relation to diagnostic age. There was an interaction between family monthly income and language development on the diagnostic age (interaction *p* < 0.001). The worse language development was related to the earlier diagnostic age of autistic children with below 5,000 yuan of family monthly income (Crudeβ = −0.57, 95%CI = −0.96–−0.18), 5,000 to 10,000 yuan of family monthly income (Crudeβ = −0.81, 95%CI = −1.01–−0.61), more than 10,000 yuan of family monthly income (Crudeβ = −0.93, 95%CI = −1.12–−0.73). Only in autistic children with below 5,000 yuan of family monthly income, the CARS score was positively related to the diagnostic age (Crudeβ = 0.06, 95%CI = 0.00−0.11), and DQ/IQ scores were negatively related to the age of diagnosis for autistic children (Crudeβ = −0.03, 95%CI = −0.04–−0.01), and adaptive function was negatively related with the age of diagnosis for autistic children (Crudeβ = −0.14, 95%CI = −0.24–−0.05).

**Table 6:** Stratified analyses according to autistic clinical phenotypes and family monthly income with diagnostic age.

**Characteristic**	**Interaction *p*-value**	<**5,000 yuan**		**5,000–10,000 yuan**		>**10,000 yuan**	
		**Adjusted** β **(95% CI)**	***p*** **-value**	**Adjusted** β **(95% CI)**	***p*** **-value**	**Adjusted** β **(95% CI)**	***p*** **-value**
CARS	0.558	0.06 (0.00–0.11)	0.050	−0.02 (−0.07–0.04)	0.541	−0.01 (−0.06–0.05)	0.788
DQ/IQ score	0.099	−0.03 (−0.04–−0.01)	0.001	−0.01 (−0.02–−0.00)	0.147	−0.00 (−0.01–0.01)	0.911
Adaptive function	0.123	−0.14 (−0.24–−0.05)	0.002	−0.03 (−0.1–0.05)	0.509	−0.02 (−0.11–0.07)	0.685
Language	< 0.001	−0.57 (−0.96–−0.18)	0.004	−0.81 (−1.01–−0.61)	< 0.001	−0.93 (−1.12–−0.73)	< 0.0001
Regression	0.754	−0.06 (−0.55–0.43)	0.817	−0.14 (−0.5–0.23)	0.467	−0.13 (−0.52–0.27)	0.5238

OR, odd risk; CI, confidence interval; DQ, developmental quotient; IQ, intelligence quotient; CARS, Childhood Autism Rating Scale.

Adjust for child sex, parity, maternal age at birth, maternal education.

Tables 7, 8 present the interview information about the first concern/referral channels from autistic parents or guardians. A total of 797 autistic parents or guardians were interviewed. More than half of autistic children (58.09%) were referred for medical care because of deficits in verbal communication. Approximately 23.59% of autistic children were referred for medical care because they impaired social interactions. The autistic children (83.06%) were almost concerned to be referred for medical care by their parents or guardians. In addition, referral channels also included medical personnel (7.90%), educational institutions (4.64%), relatives/friends/strangers (3.14%), and internet/social media (1.25%).

**Table 7:** Interview about the first concern and referral reasons from autistic parents or guardians.

**The first concern and referral reasons**	***n***	**%**
Deficits in verbal communication	463	58.09
Impairments of social interaction	188	23.59
Restrictive behaviors	18	2.26
Sensory disturbances and motor abnormalities	29	3.64
Emotional problems	11	1.38
Regression	18	2.26
Hyperactivity	14	1.76
Poor academic performance	29	3.64
Others	27	3.39

**Table 8:** Interview about referral channels from autistic parents or guardians.

**Referred group**	***n***	**%**
Parents/guardians	662	83.06
Medical personnel	63	7.90
Educational institutions (kindergarten/primary school etc.)	37	4.64
Relatives/friends/strangers	25	3.14
Internet/social media	10	1.25

**Figure 1:**
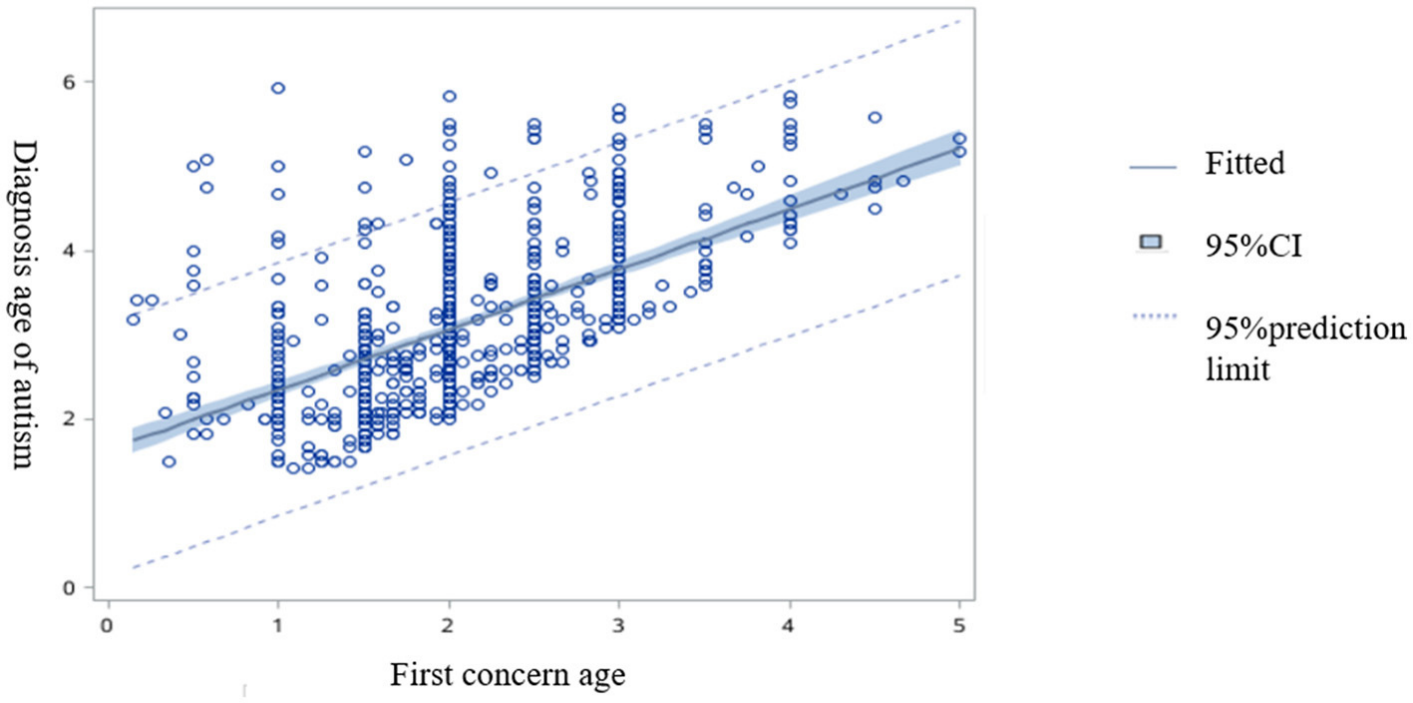
The correlation fitting plot between the first concern age from the autistic children and the diagnostic age of autistic children.

Figure 1 presents the relationship between the first concern age of the autistic children and the diagnostic age of the autistic children (β = 0.71, 95%CI: 0.64–0.79).

## Discussion

The study aimed to explore the association between clinical signs and the first concern and the diagnosis of children with autism in southern China, examining the relationship between autistic heterogeneous symptoms and the age of first concern and diagnosis and the effect modified by child sex, family socioeconomic background, and maternal educational status. We mainly found that (1) language delay was more significantly related to the age at the first concern and the diagnosis, compared with the severity of autism and other symptoms, (2) autistic children tended to be diagnosed earlier if they had an abrupt or gradual regression in social or language dimensions, (3) the effect of language on the age at diagnosis was moderated by the child's sex, the family's socioeconomic status, or the education status of the child's mother. Language delay was associated with an earlier age at diagnosis more strongly among boys than girls. Compared to the lower maternal educational level or family income level, language delay was most associated with an earlier age at diagnosis for autistic children with a high maternal educational level or family income status. (4) The effect of cognitive or adaptive development on the age at diagnosis was moderated by the family's socioeconomic status or the education status of the child's mother. Cognitive development, or adaptive development delay, was significantly related to a later age at diagnosis in autistic children whose mothers had a low education status or a low family income.

Our study showed that autistic children with worse language impairments were diagnosed earlier. Our finding was consistent with those of previous studies, which proposed that verbal and non-verbal communication deficits, rather than social interaction difficulties or restricted repetitive behaviors, were crucial for the early determination of autism (61, 62). Language deficits are an important component of the diagnosis of autistic children. The public may be most familiar with the symptom of language delay from the description of autism in the media. Parents often expect normal language development for children in the first 2 years of life. After reaching the age of 2 years old, children often transition from simpler to more complex social situations. The language deficits in children may be more confusing for parents, accelerating additional concerns and early diagnosis (62). Our study also demonstrated that the effect of language delay on the age of diagnosis was moderated by child sex, such that language delay was associated with an earlier diagnostic age more strongly among boys. Several studies were consistent with the ideas of our study, which demonstrated that girls were more likely to receive a later diagnosis of autism than boys (63). The disorder of autism was always deemed to disproportionately affect males and portrayed the conception of “male disorder.” Compared with males, autistic females tended to have more severe symptoms or suffer from other neurodevelopmental diseases before being diagnosed with autism (63). Halladay et al. (64) point out that females may display autistic symptoms more subtly than males and that they may be more likely to cover the underlying symptoms, which may lead to greater challenges in diagnosing females with autism. Meanwhile, parents may react differently to the communication problems of boys and girls. Boys may be more concerned due to higher parental expectations of them. The finding indicates an urgent need to raise growing awareness among clinicians and parents that girls with autism may present with subtle clinical differences when compared with boys.

In line with previous studies (40, 65), we also found that regression was associated with an earlier diagnosis of autism. Even though regression is not a diagnostic criterion for autism, concern with an abrupt or gradual loss of previously acquired skills, such as language and/or social communication, can be crucial for the early identification of autism. Autistic children who had regressive symptoms before or at 36 months received the diagnosis at a younger age than children without regressive symptoms (40, 65). There were similar impairments in early development domains and a similar amount of repetitive and stereotyped behaviors between children with regression and children without regression. The development before the regression may not be typical. A common early-onset and following regression pattern or developmental delay may already exist in the early period with later abrupt regression or gradual losses (66, 67). Early developmental impairments may not attract parents' attention because they may consider that the child is still young, the development is immature, or the child is considered to have a temporary developmental delay. The loss of previously acquired skills greatly raises parental concerns because of the developmental trajectories beyond expectations.

The study presented different views on the relationship between cognitive or adaptive development and diagnostic age. Previous studies have demonstrated that children with lower IQs may have been earlier identified as having autism. Our finding revealed that children with low cognitive or adaptive development were more likely to be diagnosed later, which was not consistent with the above opinion. After stratified analysis by maternal education status or family income, the trend remained only in children exposed to lower maternal education status or family income. Besides, among autistic children with these family characteristics, e.g., high maternal educational level or high family income level, language delay was most associated with an earlier age at diagnosis. Several reasons may be interpreted for the results. However, there has been increased awareness of autism among the public in China in the last few decades. Language deficits should be the first early warning signs of autism for parents and clinicians. Other symptoms, such as cognitive or adaptive development delays, may not be strong enough to prompt parental attention to consider autism as an initial suspicion for their child. Instead, these symptoms may increase the likelihood of mistakenly identifying the child as having other neurodevelopmental diseases. In such cases, low cognitive or adaptive development may lead parents to perceive their child as having only a developmental delay, which could hinder the early detection of autism. The above status may be further exacerbated, especially in populations with low family income, by factors such as limited access to medical knowledge and healthcare services. Lung et al. (31) found a disparity in the association between a mother's level of education and the diagnostic age of an autistic child. Mothers with a lower level of education were found to be less likely to possess comprehensive knowledge of autism symptoms compared to mothers with higher educational levels. Additionally, they faced greater challenges in accessing healthcare services, including autism diagnosis and early intervention (31). When children exhibit cognitive or adaptive developmental delays, parents with a lower educational level may often overlook the symptoms and delay consulting clinicians in a timely manner. There may exist disparities in autism diagnosis among children from different social-economic populations, primarily attributable to limitations in the availability and accessibility of health services (29). There is an urgent need to increase awareness of autism, especially among low-income groups. Parents play a critical role in recognizing potential developmental delays in their children. By actively engaging with the healthcare system, they can seek a diagnosis of autism. Increasing familiarity with autism will lead to improved diagnosis rates and earlier recognition of the condition, facilitating timely interventions and support for affected children (25).

### Interviews for parents or guardians

Our teamers also actively took part in the interview with autistic children's parents or guardians regarding the first concern reasons as well as the referral channels (Tables 7, 8). Approximately 662 autistic children were first referred by parents or guardians themselves (83.06%). Other referral channels include clinicians (7.9%), teachers (4.64%), family relatives or friends (3.14%), and the Internet/social media (1.25%). The most frequently reported initial concern prompting parents/guardians to seek medical attention for their children was the deficit in verbal communication, accounting for 463 cases (58.09%). A mother recalled her son's experience: He did not speak until he was about 1 year old. She and other family members thought it might not be trouble initially and that the symptoms would improve with age. However, as the child reached the age of 2 years or older and was still unable to speak, parents started experiencing anxiety and subsequently sought further clinical help. In addition, other symptoms, including delay in naming responses, non-playing with others, restrictive/stereotypic behaviors, and emotional problems, were also reported by parents or guardians. Approximately 188 (23.59%) autistic children's parents reported that their children had an impairment in social interaction, including delays in naming responses, non-playing with others, no eye contact, and non-following commands. One participant described the symptoms of his daughter: We found that she did not respond when we attempted to make her laugh and failed to communicate with us when she was about 16 months old, which made us feel that she was living in her world and stayed lonely by herself. After going to kindergarten, the teacher also pointed out that my little girl could not follow the instructions and was unwilling to play with others, and consequently, our family decided to attempt to seek help from clinicians. Nearly 18 (2.26%) of autistic children's parents noticed that their children had stereotyped repetitive behavior as the first symptom, such as repeatedly opening and closing the door and being interested in looking at the fan or air conditioner. In addition, sensory disturbances and motor abnormalities (29/3.64%), emotional problems (11/1.37%), regression (18/2.24%), and hyperactivity (14/1.76%) were also reported by autistic children's parents. In this study, we also found that their first concern about the possible neurodevelopmental abnormalities of their children was associated with the diagnostic age of autism. There is an urgent need to further educate parents about developmental milestones and autism.

### Strengths and limitations

There are several strengths. First, this is a longitudinal autistic cohort study with a large sample size to explore the clinical profiles of autism, the age of diagnosis, and the age of parents' first concerns. Second, the autistic children were strictly diagnosed by clinicians based on the DSM-5 and interviewed according to the Autism Diagnostic Observation Schedule (ADOS) and the Autism Diagnostic Interview-Revised (ADI-R). Third, the study further found that the association between clinical profiles of autism and the age of diagnosis is mediated by child sex or family sociodemographic factors. Several limitations should be noted in this study. First, it was conducted exclusively within the Chinese population, which may restrict the generalizability of the findings to other ethnic populations. Second, there is a possibility of recall bias from parents/guardians who provided information. Third, as the study was observational in nature, there might be other residual confounding factors that could influence the results.

## Conclusion

This cohort study found that language delay and regression were the priority symptoms associated with age of first concern and diagnosis among children with autism spectrum disorder in southern China. Language delay was related to an earlier age at diagnosis more strongly among autistic boys than autistic girls. Cognitive development, or adaptive development delay, was correlated with a later age at diagnosis in autistic children whose parents had a low education status or a low family income. The female sex, low education status, or low family income were identified as key factors relating to disparities in the identification of autism. The present results highlight that there is an urgent need for further comprehensive advocacy for autism, especially for early concern autistic clinical signs such as language deficits and possible key sociodemographic factors so that parents/guardians and clinicians in relevant fields can improve the awareness of early identification of autism and finally shorten the span between the age of first concern and the age of diagnosis.

## Data availability statement

The raw data supporting the conclusions of this article will be made available by the authors, without undue reservation.

## Ethics statement

The studies involving humans were approved by Guangzhou Women and Children's Medical Center Ethics Committee. The studies were conducted in accordance with the local legislation and institutional requirements. Written informed consent for participation in this study was provided by the participants' legal guardians/next of kin. No potentially identifiable images or data are presented in this study.

## Author contributions

W-XC: concept and design, integrity of the data, accuracy of the data analysis, and critically revised. XL: draft the manuscript. XL and CG: statistical analysis. All authors contributed to the collection and interpretation of the data.
